# Climate‐Driven Habitat Suitability Modeling for the Vulnerable Species *Euryops pinifolius* A. Rich in Ethiopia: Implications for Conservation

**DOI:** 10.1002/ece3.73566

**Published:** 2026-04-30

**Authors:** Liyew Birhanu, Heiko Balzter, Laura Basell, Moya Burns, Wale Arega, Yilkal Gebeyehu

**Affiliations:** ^1^ School of Life Sciences and the Environment, Royal Holloway, University of London Egham Surrey UK; ^2^ School of Geography, Geology and the Environment, Institute for Environmental Futures, University of Leicester Leicester UK; ^3^ Department of Biology Debre Markos University Debre Markos Ethiopia; ^4^ National Centre for Earth Observation University of Leicester Leicester UK; ^5^ School of Heritage and Culture, Institute for Environmental Futures, University of Leicester Leicester UK; ^6^ School of Biological and Biomedical Sciences, Institute for Environmental Futures, University of Leicester Leicester UK; ^7^ Department of Natural Resource Management Debre Markos University Debre Markos Ethiopia

**Keywords:** climate change, conservation planning, Ethiopia, *Euryops pinifolius*, habitat suitability, MaxEnt model

## Abstract

Globally, rare and threatened species are a high conservation priority due to their increased risk of extinction. *Euryops pinifolius* is a vulnerable plant species found within restricted ranges in the Afroalpine Ecosystem of Ethiopia. The species has been seriously threatened due to overexploitation for fuelwood collection by the communities living adjacent to the mountains. Given the socio‐economic importance as well as the increasing vulnerability of the species, it is important to understand how extinction may be prevented in the face of climate change. The present study, therefore, aimed to model and map habitat suitability of the species under climate change scenarios in Ethiopia. In this study, we used the MaxEnt model to predict the current and future distribution of *E. pinifolius* in Ethiopia under different climate scenarios. The results demonstrated excellent simulation quality by the MaxEnt model (AUC = 0.985). The major environmental factors predicting the current and future distribution of *E.pinifolius* were *Mean Temperature of the Driest Quarter* (Bio9), *Altitude* (Alt), *Vegetation Cover* (Veg), *Precipitation Seasonality* (Bio15), and *Mean Temperature of the Wettest Quarter* (Bio8). Under current climatic conditions, the potential distribution of *E. pinifolius* is primarily concentrated in the Ethiopian highlands, especially in the northern, northwestern, and central parts of the country. We therefore recommend that both current and projected future suitable areas be given conservation priority to safeguard *this species.*

## Graphical abstract

*Euryops pinifolius*, vulnerable plant species in Ethiopia's Afroalpine ecosystem, faces serious threats from overexploitation for fuelwood. Using the MaxEnt model (AUC = 0.985), this study identified key environmental factors‐such as temperature, altitude, vegetation cover, and precipitation seasonality‐affecting its current and future habitat suitability. The species' suitable habitats are mainly in the northern, northwestern, and central Ethiopian highlands, and these areas should be prioritized for conservation under current and future climate scenarios.

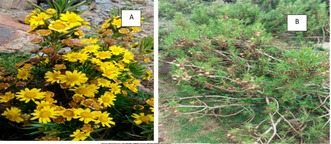

## 1 Introduction

Biodiversity loss is an urgent global concern, with numerous species disappearing due to anthropogenic activities (Sala et al. 2000; Pereira et al. 2010; Johnson et al. 2017; IPBES 2019). Land‐use change, habitat fragmentation, overexploitation, pollution, invasive species, and climate change are widely recognized as major drivers of this problem (Fahrig 2003; Watson et al. 2005; Wilson et al. 2016; Newbold 2018; IPCC 2021). However, the relative importance of these stressors is often context‐specific, and their interactions can be complex and poorly understood (Nešić and Bjedov 2021). In many ecosystems, addressing more immediate and tangible pressures, such as fragmentation and land‐use conversion, may offer more achievable entry points for conservation and mitigation efforts, while acknowledging that broader forces, such as climate change, exacerbate these underlying challenges (Muluneh 2021). Numerous species are therefore facing extinction, driven by the combined influence of these human‐induced changes (Tilman et al. 2017).

Likewise, plant distribution patterns are influenced by environmental factors such as climate, soil nutrients, human disturbances, and topographic features, which vary across different regions (Liu et al. 2003; Pugnaire et al. 2012; Brown et al. 2019). Global climate change poses significant risks to species and ecosystems, causing shifts in species distribution (Chen et al. 2011), threatening their viability via range reduction (Thomas et al. 2004), and altering the representation of species in protected areas (Velásquez‐Tibatá et al. 2012). Species confined to mountain tops are at significant risk of extinction, as they may not have enough suitable habitats they can migrate to in response to climate change (Lenoir et al. 2008; Markham 2014). Consequently, understanding the impact of climate change and other anthropogenic pressures on plant distribution is essential for effective conservation planning and management (Urban et al. 2016; Charney et al. 2021).

The Horn of Africa is one of 36 global biodiversity hotspots, incorporating many endemic and threatened plants, yet climate impact studies in this region are scarce (Myers et al. 2000; Mittermeier et al. 2005). Africa will face temperature rises of 3°C–4°C between 2080 and 2099, approximately 1.5 times higher than the global mean (IPCC 2021). Climate change poses a threat to a substantial portion of tropical African flora, with many species potentially facing extinction (Stévart et al. 2019). These impacts are expected to drive major shifts in species distributions, particularly affecting endemic and montane ecosystems, and to increase the risk of habitat loss across the continent (Warren et al. 2013; Midgley and Bond 2015). Specifically, it is estimated that over 5000 African plant species will lose their climatically suitable habitats by 2085 (McClean et al. 2005; Yebeyen et al. 2022). Mapping and monitoring species' responses to ongoing change for effective management is therefore essential.

Of 6027 plant species recorded in the Flora of Ethiopia and Eritrea, 647 (10.74%) are endemic, and many of these cluster in the Afroalpine and sub‐Afroalpine regions (Friis et al. 2010; Demissew et al. 2021). Threats to endemic Ethiopian species vary, as demonstrated by studies of *Lobelia rhynchopetalum*, which is at risk of extinction due to climate warming, and *Echinops kebericho*, which is heavily exploited for its medicinal value (Vivero et al. 2005; Chala et al. 2016; Tafesse et al. 2023).

*Euryops pinifolius* A. Rich. (Family: Asteraceae) is native to Ethiopia, grows primarily in the seasonally dry tropical biome, and was chosen for our study based on its threatened status and narrow distribution ranges, making it ideally suited for modeling (Vivero et al. 2005). *Euryops pinifolius* is a shrub found in montane meadows at altitudes of 3200–3700 m. asl often alongside genus lobelia on thin soil, rocks, and cliff margins. It is economically valued by local communities for its medicinal uses and as fuelwood (Kelbessa et al. 1992). Medicinal qualities include pain relief by chewing the leaf, especially for stomachache (kurba), and it has been demonstrated to have antioxidant properties (Meragiaw et al. 2016; Rehman et al. 2022). In some regions of Ethiopia, it is the most collected firewood species because it can be easily burned without drying (Ashenafi 2001). Parts of the plant are also used for feeding livestock and for ornamental purposes (esthetics and recreational values) (Guassa Area General Management Plan (GMP) 2005).

The International Union for Conservation of Nature (IUCN) has listed the plant as vulnerable due to anthropogenic impacts on its habitat, leading to legislation restricting harvesting in some areas (Vivero et al. 2005; Steger et al. 2020). To determine the best conservation strategies for *E*. *pinifolius*, an understanding of the environmental factors that affect its geographical distribution is required. Despite the clear importance of this shrub, the effects of global climate change on its distribution remain unclear.

Among the most important research problems for ecologists is understanding how species interact with environmental factors to predict how these factors will change species distributions (Cao et al. 2016). An important consideration in predicting the habitat suitability of species under climate change is that it provides critical information for the conservation of endemic and threatened plant species. Tools include species distribution models (SDMs) for investigating habitat suitability, identifying environmental variables driving species patterns, and identifying areas where urgent conservation measures are needed (Elith et al. 2011; Oyebanji et al. 2021).

A wide range of species distribution modeling techniques is available, with varying performance and data type requirements (Elith et al. 2006). Among the best algorithms, MaxEnt (Maximum Entropy) is one of the most useful techniques for modeling endemic, rare, and threatened species with limited habitat ranges and scant presence‐only occurrence data (Phillips and Dudík 2008; Dyderski et al. 2018; Chen et al. 2022). In this study, the MaxEnt model was employed to predict potentially suitable habitats for *E. pinifolius* under various climate change scenarios in Ethiopia. The objectives of this research are to: (a) estimate the current and future habitat suitability for *E. pinifolius* under these climate change scenarios; (b) identify the significant environmental factors influencing the geographical distribution of *E. pinifolius*; and (c) recommend priority areas for effective future conservation of the species.

## 2 Materials and Methods

### 2.1 Species Occurrence Data

**FIGURE 1:**
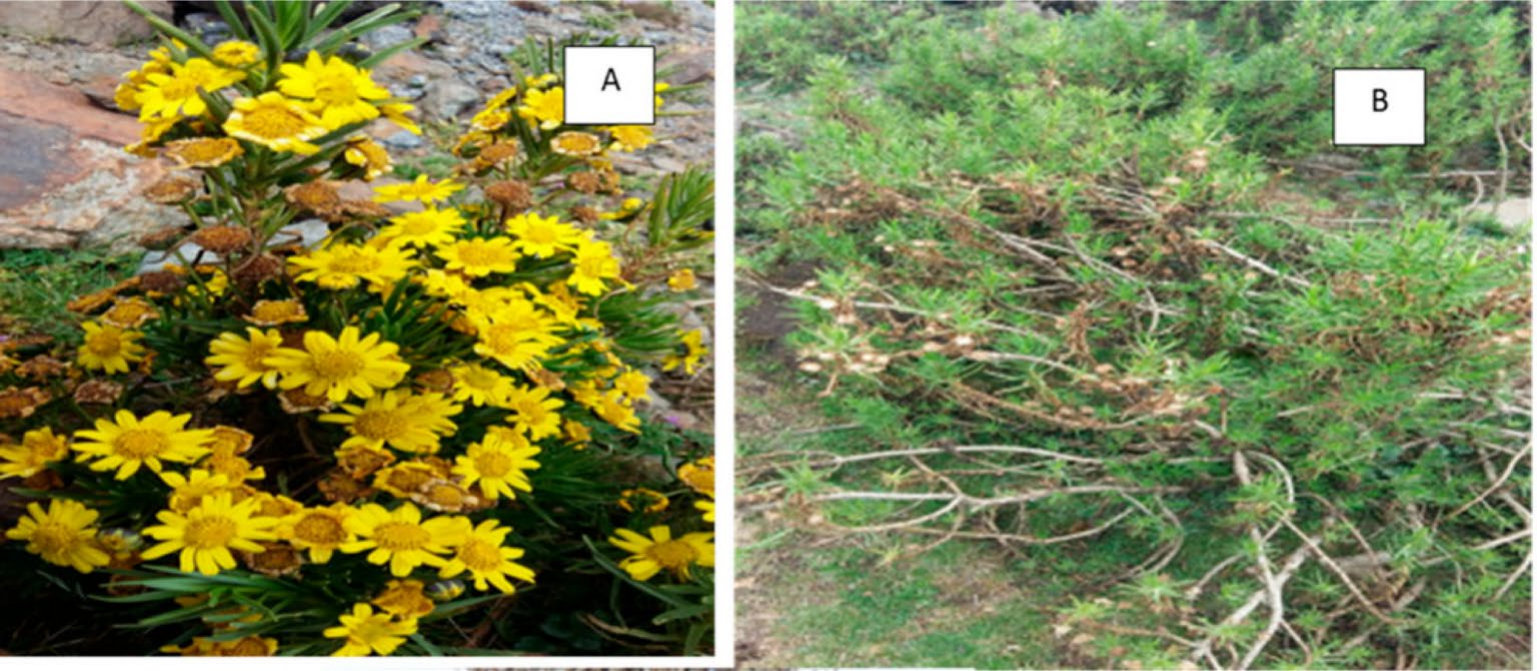
*Eyuryops pinifolius* A. Rich (A) flowering stage (B) seed dispersal stage (Dagnachew et al. 2023).

**FIGURE 2:**
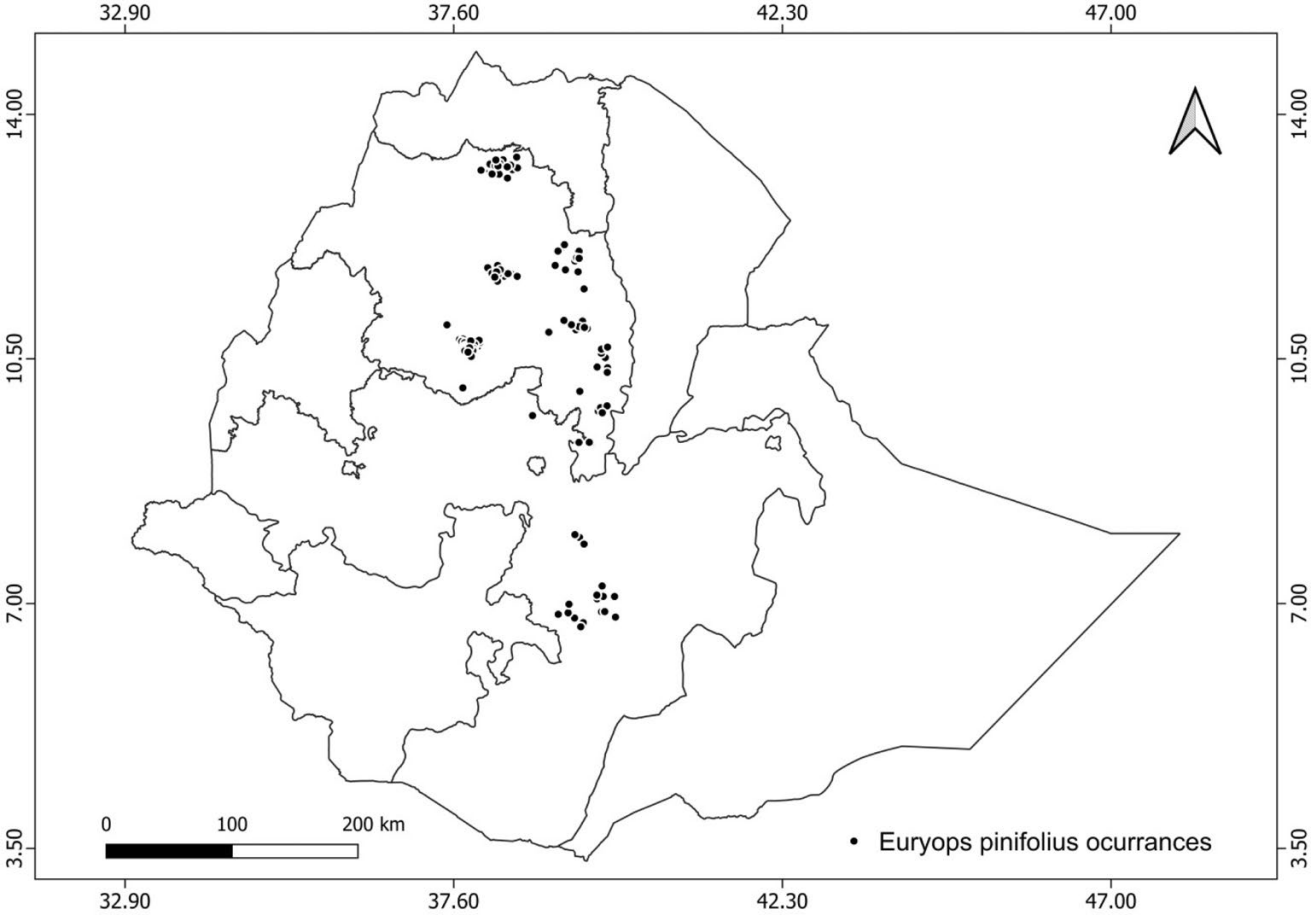
The location of 151 occurrence points of *E. pinifolius* in Ethiopia.

*E. pinifolius* A. Rich is a member of Asteraceae, a shrub that grows between 3200 and 3700 m above sea level in the Sub‐Afroalpine vegetation of Ethiopia (Vivero et al. 2005). It grows on the edges of cliffs, on rocks, and in thin soil alongside Lobelia. We obtained 151 occurrence points for *E. pinifolius* from the Global Biodiversity Information Facility (https://www.gbif.org), Addis Ababa University's National Herbarium of Ethiopia, field surveys, and previously published literature (Table S1). To ensure the accuracy of occurrence data, a rigorous multi‐step cleaning and rarefaction process was applied. Species names and geographic coordinates were carefully verified using herbarium records, ArcGIS, and Google Earth. To reduce spatial autocorrelation, occurrence points were spatially rarefied at a 1km^2^ resolution using SDM Toolbox v2.6 (Brown 2014; Guisan et al. 2017). Additionally, spatial thinning was conducted to remove duplicate records (Aiello‐Lammens et al. 2015). Finally, a total of 136 occurrence points for *E. pinifolius* were retained for the final analysis to generate the potential distribution using MaxEnt modeling. The flowering and seed dispersal stages of *E. pinifolius* are shown in Figure 1. The occurrence‐point data were saved in a CSV file, organized by species name, longitude, and latitude, and subsequently plotted on a map (Figure 2).

### 2.2 Environmental Variables

To determine the habitat suitability of *E. pinifolius*, 19 bioclimatic variables, 3 biophysical factors (elevation, slope, and aspect), as well as land use/land cover, and vegetation data were used. We obtained climate data for the current period (1970–2000) and future bioclimatic projections for the 2050s (2041–2060) and 2070s (2061–2080) from WorldClim 2.1 (https://www.worldclim.org/), with a spatial resolution of 30 arc sec (approximately 1 km^2^). Two shared socioeconomic pathways (the worst SSP8.5 and the intermediate emission pathway SSP4.5) developed by HadGEM2‐Es global circulation models (GCMs) were used during the 2050s (2041–2060) and 2070s (2061–2080) (Fick and Hijmans 2017). Topographic variables were derived from the Shuttle Radar Topography Mission Digital Elevation Model (SRTM‐DEM) (Jarvis et al. 2008).

Ethiopian vegetation types were derived from the World Agroforestry Centre (http://landscapeportal.org/layers/geonode:veg), whereas the land use land cover map was obtained from (https://cds.climate.copernicus.eu/). Using spatial analyst tools in ArcGIS version 10.7 at a resolution of 1 km^2^, all environmental variables were processed to have the same extent, projection, and resolution (Phillips et al. 2006). The environmental data were then converted into “asc” files, which are necessary for Maxent modeling, using the ArcGIS software. To reduce multicollinearity among the 24 environmental variables, highly correlated variables (*r* ≥ 0.70, Pearson correlation coefficient) were eliminated from further models (Guisan et al. 2017). Finally, 13 environmental variables were used to model the geographical distribution for *E. pinifolius* (Table 1).

**TABLE 1:** List of Environmental variables (13 variables with the code shown in bold font were chosen for the MaxEnt modeling study). Capitalized codes (e.g., Alt, ASP, Slope, LULC, Vegetation) represent topographic and land‐cover variables, while Bio1–Bio19 represent bioclimatic variables.

Code	Environmental variables	Unit
Bio1	Annual mean temperature	°C
Bio2	Mean diurnal range	°C
Bio3	Isothermality	—
**Bio4**	**Temperature seasonality**	—
**Bio5**	**Maximum temperature of the warmest month**	°C
Bio6	Minimum temperature of the coldest month	°C
Bio7	Temperature annual range	°C
**Bio8**	**Mean temperature of the wettest quarter**	°C
**Bio 9**	**Mean temperature of driest quarter**	°C
Bio10	Mean temperature of the warmest quarter	°C
Bio11	Mean temperature of the coldest quarter	°C
Bio12	Annual precipitation	mm
Bio13	Precipitation of the wettest month	mm
**Bio14**	**Precipitation of the driest month**	mm
**Bio15**	**Precipitation seasonality**	—
Bio16	Precipitation of the wettest quarter	mm
Bio17	Precipitation of the driest quarter	mm
**Bio18**	**Precipitation of the warmest quarter**	mm
**Bio19**	**Precipitation of the coldest quarter**	mm
**Alt**	**Altitude**	M
**ASP**	**Aspect**	Degree
**Slope**	**Slope**	%
**LULC**	**Land use Land cover**	%
**Vegetation**	**Vegetation cover**	%

### 2.3 Species Distribution Modeling

**FIGURE 3:**
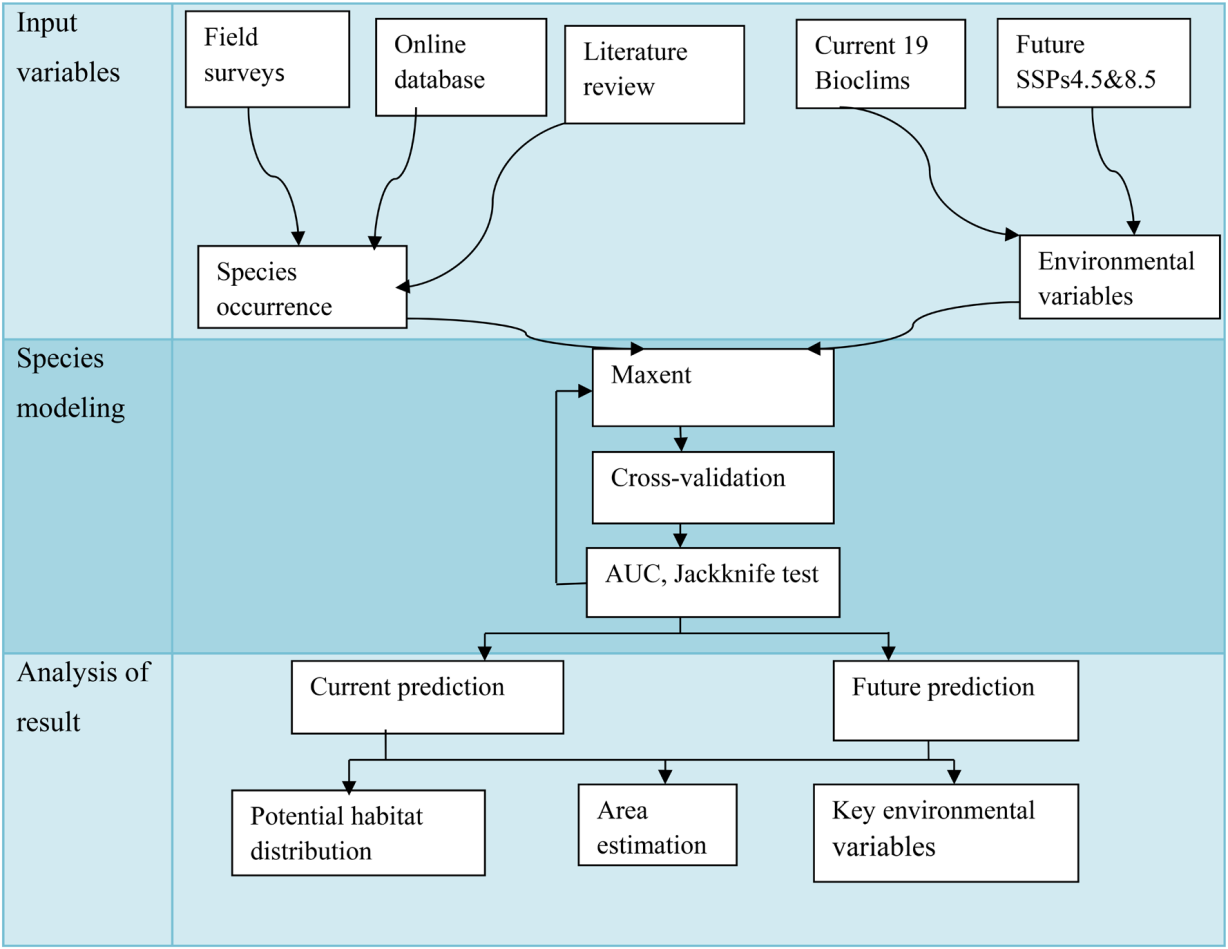
Flow chart diagram methodology used in species distribution modeling.

We employed the maximum‐entropy algorithm (Maxent version 3.4.1) species distribution modeling algorithm to predict the current and future habitat suitability of *E. pinifolis*. In the modeling, 75% of the data was used for training the model, whereas 25% of the data was used for model testing (Phillips et al. 2006). The performance of the MaxEnt models was evaluated using the area under the curve (AUC) of the receiver operating characteristic (ROC) value. The AUC values range from 0 to 1, where high AUC values imply a good model fit. The model's predictions become more accurate, and confidence increases as the AUC value approaches 1 (Swets 1988). Therefore, models with an AUC > 0.90 are generally considered to be highly accurate, while those with 0.70 < AUC ≤ 0.90 are viewed as good. AUC values between 0.50 and 0.70 suggest lower accuracy, and an AUC ≤ 0.50 indicates performance no better than random chance (Swets 1988; Elith et al. 2011). Jackknife analyses were performed to determine variables that significantly influence the model reliability. Referring to the classification proposed by Gao et al. (2022), four habitat classes were generated: unsuitable habitat (0–0.20); lowly suitable habitat (0.21–0.50); moderately suitable habitat (0.51–0.70); highly suitable habitat (> 0.70). The area for optimal distribution, classified as low, medium, and high suitable habitats, was calculated for each model. The overall methodological framework of the study is illustrated in Figure 3.

## 3 Results

### 3.1 Model Performance and Accuracy

**FIGURE 4:**
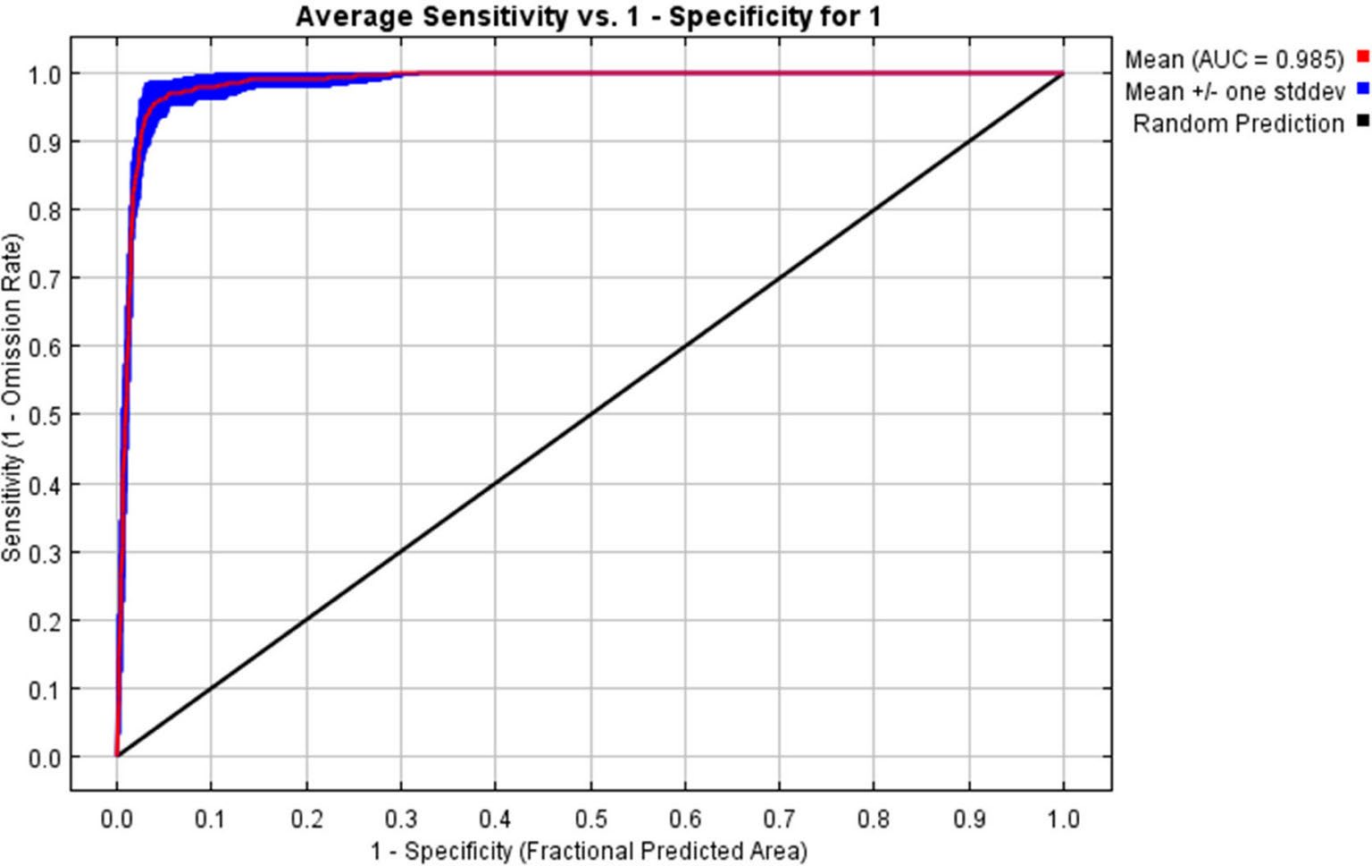
The area under the receiver operating characteristic curve for *E. pinifolius* obtained via MaxEnt modeling.

Our model demonstrated excellent performance, achieving an average AUC value of 0.985. This indicates that the MaxEnt model was highly reliable in predicting the potential geographical distribution areas of *E. pinifolius* in Ethiopia (Figure 4).

### 3.2 Environmental Variable Contributions for *Euryops pinifolius* Distribution in Ethiopia

The MaxEnt analyses identified key environmental variables that contributed to the distribution model of *E. pinifolius* in Ethiopia under different climate change scenarios. Mean Temperature of the Driest Quarter (Bio9) had the highest contribution in the model, followed by Altitude (Alt), Vegetation Cover (Veg), Precipitation Seasonality (Bio15), and Mean Temperature of the Wettest Quarter (Bio8) (Table 2).

**TABLE 2:** Relative percent contribution of environmental factors to MaxEnt modeling for *Euryops pinifolius* distribution in Ethiopia.

Variable	Percent contribution				
	Current	2050		2070	
		SSP4.5	SSP8.5	SSP4.5	SSP8.5
Bio 9	46.4	49.71	43.88	46.09	40.99
Alt	36.86	30	38.32	24.87	38.69
Veget	4.90	5.67	6.30	5.76	5.82
Bio8	3.24	4.65	2.61	13.86	2.14
Bio15	2.85	3.6	3.2	3.47	2.78
Bio5	2.11	3.33	1.46	2.71	6.31
Bio19	1.08	1.45	1.33	1.39	1.34
LULC	0.728	0.44	0.74	0.561	0.46
Slope	0.63	0.42	0.78	0.44	0.30
Bio18	0.56	0.43	0.39	0.30	0.57
ASP	0.52	0.25	0.57	0.25	0.34
Bio4	0.25	0.40	0.27	0.21	0.14
Bio14	0.06	0.06	0.08	0.03	0.06

**FIGURE 5:**
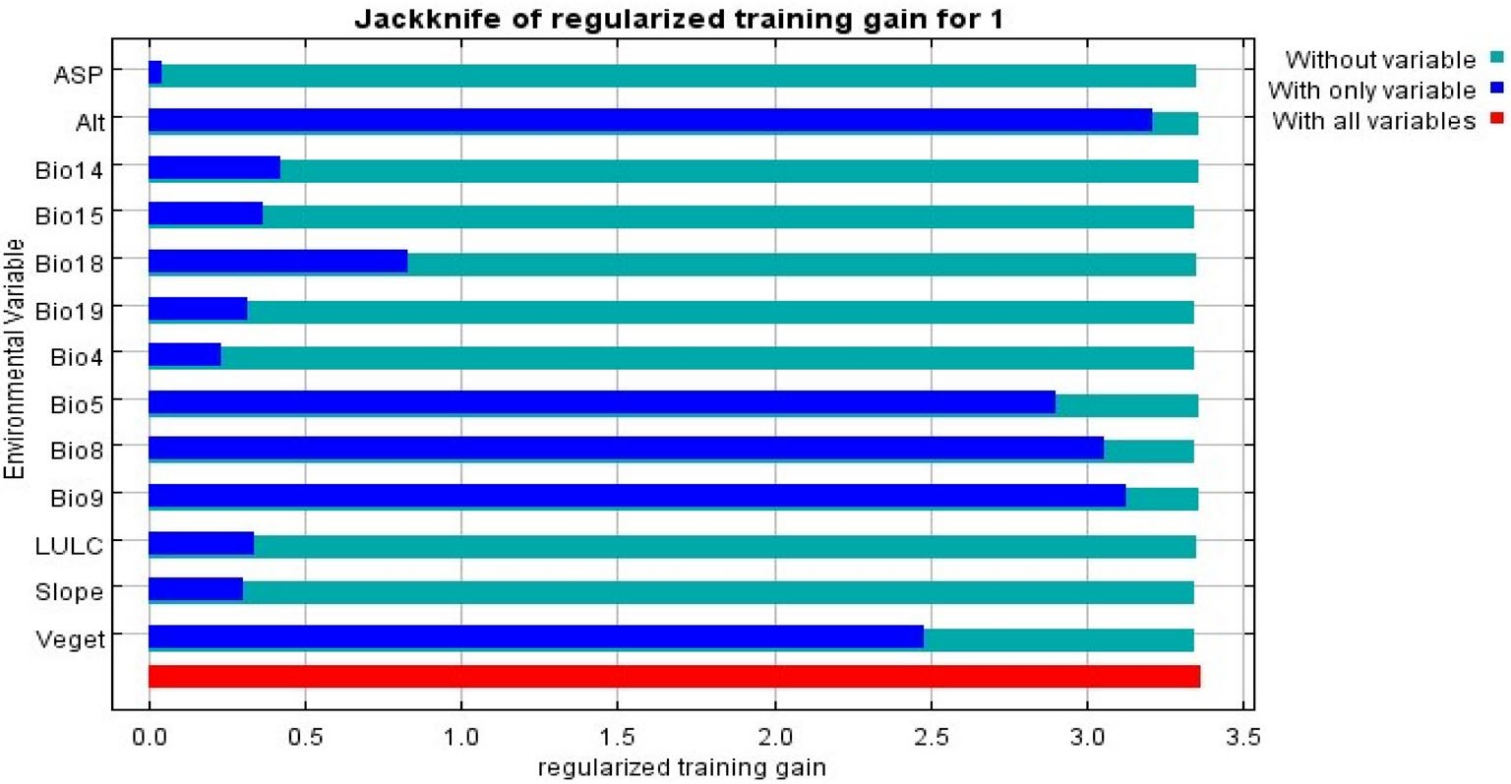
Predictive power of different environmental variables based on the jackknife of regularized training gain.

**FIGURE 6:**
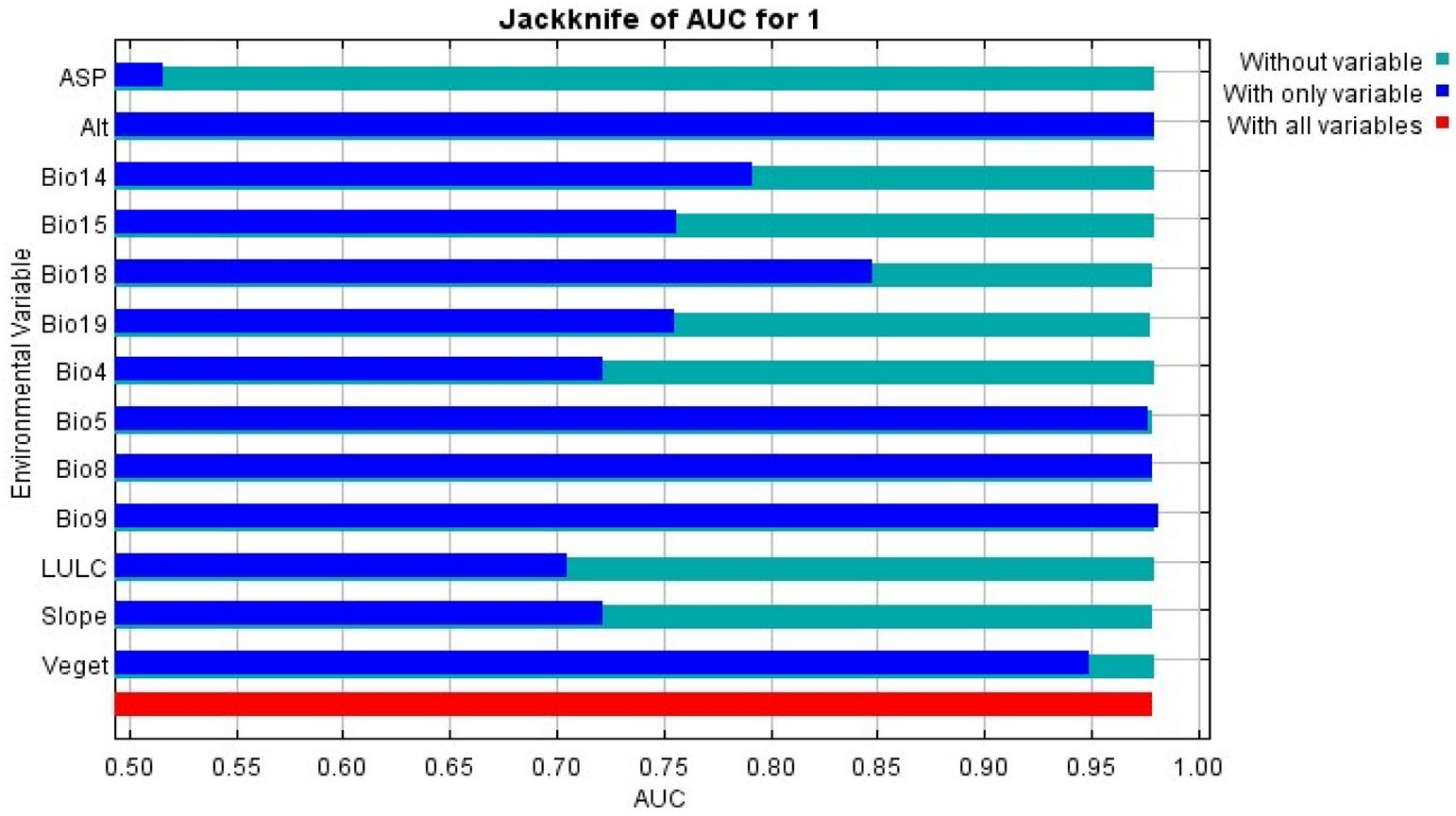
Result of jackknife AUC test of the MaxEnt model for evaluating the relative importance of bioclimatic environmental variables for *E. pinifolius*.

The jackknife analysis also revealed that altitude (ALT) and the Mean Temperature of the Driest Quarter (Bio9) were the most influential environmental variables when used individually, indicating their important role in predicting the geographical distribution of *E. pinifolius* (Figure 5). Other significant contributors included Bio8 (Mean Temperature of Wettest Quarter), Bio5 (Maximum Temperature of Warmest Month), vegetation cover, Bio18 (Precipitation of Warmest Quarter), Bio14 (Precipitation of Driest Month), Bio15 (Precipitation Seasonality), land cover, and slope. In contrast, aspect showed a negligible impact on the model's predictions (Figures 5 and 6).

### 3.3 Response of *E. pinifolius* A. Rich to the Main Contributing Environmental Variables

**FIGURE 7:**
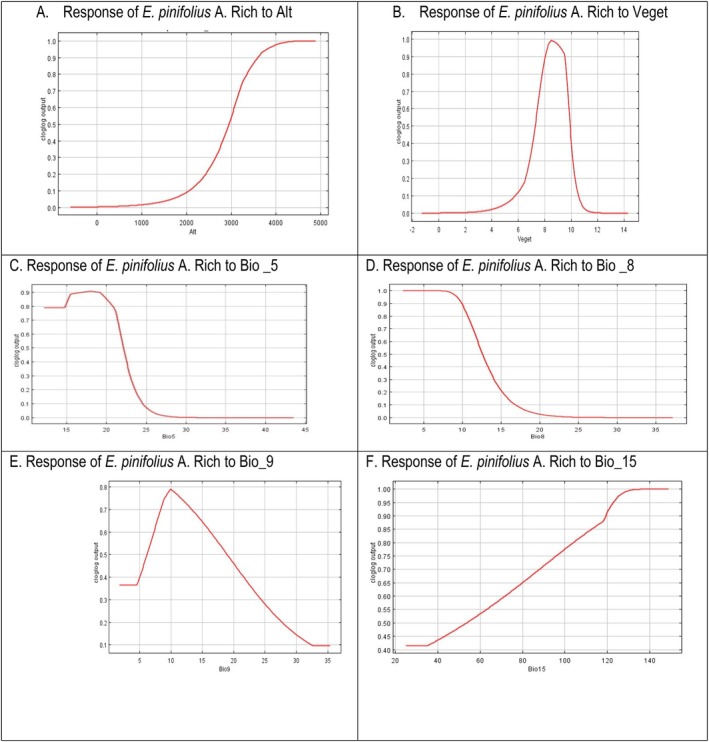
Response curve of the major bioclimatic and topographic factors influencing the distribution of *E*. *pinifolius* A. Rich (A‐F). These key variables showed an initial increase in suitability, followed by a decline as each variable approached its optimal range. Specifically, Alt showed the highest suitability at an elevation range between 3000 and 4000 m, Mean Temperature of the Driest Quarter (BIO9) was most favorable at 10°C.

The impact of each predictor variable on the MaxEnt model projection was demonstrated by the response curves (Figure 7). Each curve is a distinct model generated with the help of the corresponding variable. A MaxEnt model constructed using just the corresponding variable is represented by each curve listed below. These graphs show how predicted appropriateness depends on the chosen variable and on dependencies arising from correlations between the chosen variable and other factors.

### 3.4 Current and Future Habitat Suitability of *Euryops pinifolius* in Ethiopia

**FIGURE 8:**
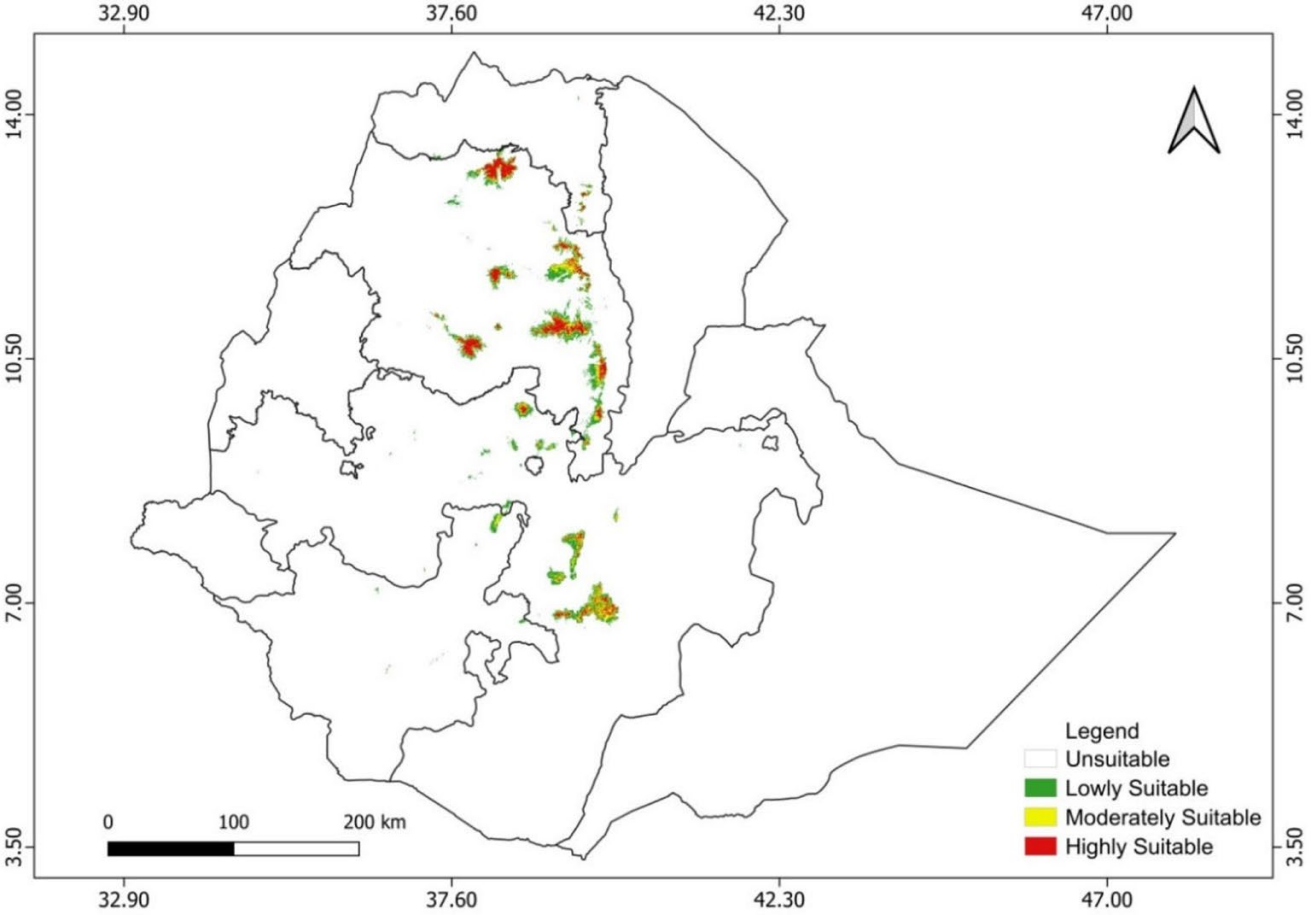
Current potential suitable habitats of *Euryops pinifolius* in Ethiopia.

The current potential distribution of *E. pinifolius* is primarily concentrated in specific highland and mountainous regions in the central, northern, and northwestern parts of the country, including the Amhara and Tigray regions, as well as parts of Oromia and Southern Nations, Nationalities, and Peoples' Region (SNNPR) (Figure 8). The total suitable area accounted for 13,420.48 km^2^. Of this, 9310.06 km^2^ is classified as lowly suitable habitat, 4110.42 km^2^ as moderately suitable habitat, and 4327.75 km^2^ as highly suitable habitat (Table 3, Figure 8).

**TABLE 3:** Suitable areas for the distribution of *E. pinifolius*.

Period	SSP	Classes of suitable habitats							
		Highly suitable		Moderately suitable		Lowly suitable		Unsuitable	
		Area (km^2^)	%	Area (km^2^)	%	Area (km^2^)	%	Area (km^2^)	%
Current	—	4327.75	0.38	4110.42	0.36	9310.06	0.82	1,124,629.22	99
2050	4.5	871.881	0.08	4283.26	0.38	12,334.69	1.1	1,124,887.62	98.9
	8.5	4680.27	0.82	3823.79	0.33	8987.49	0.78	1,124,885.90	98
2070	4.5	4972.89	0.43	3952.99	0.34	10,128.89	0.89	1,123,322.68	99
	8.5	927.497	0.08	4602.41	0.4	12,143.89	1.07	1,124,703.66	99

**FIGURE 9:**
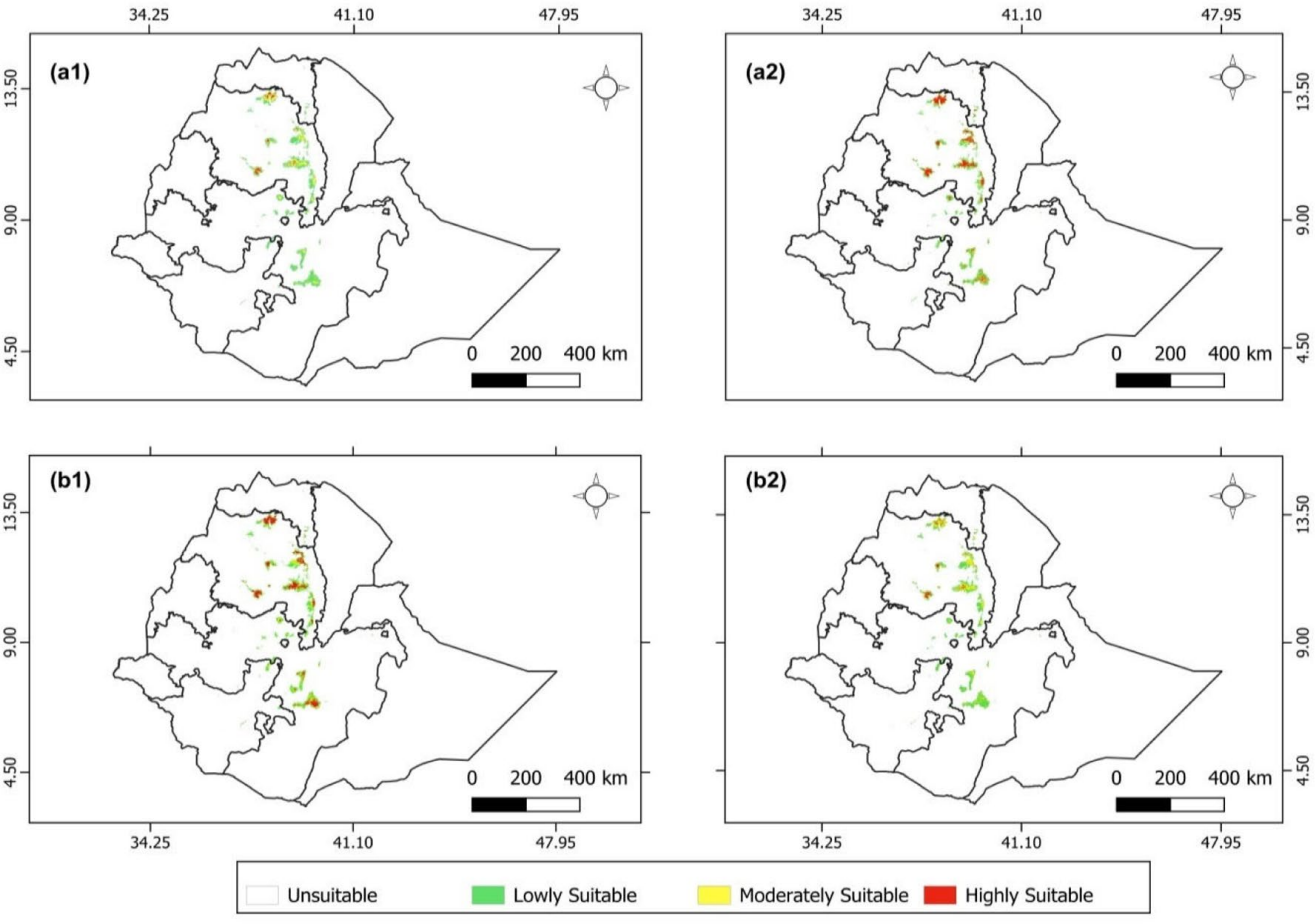
Maps indicating the predicted potential suitable habitat distribution for *Euryops pinifolius* under different climate scenarios: a1: SSP4.5_250; a2: SSP8.5_250; b1: SSP4.5_270; b2: SSP8.5_270. The a1 map (SSP4.5_250) shows habitat suitability by 2050 under the intermediate emission scenario, a2 (SSP8.5_250) shows habitat suitability by 2050 under the worst‐case emission scenario, b1 (SSP4.5_270) shows habitat suitability by 2070 under the intermediate emission scenario, and b2 (SSP8.5_270) shows habitat suitability by 2070 under the worst‐case emission scenario.

For 2050, under the SSP 4.5 scenario, the total suitable area for *E. pinifolius* increases to 17,489.83 km^2^, with 12,334.69 km^2^ classified as low suitability, 4283.26 km^2^ as moderately suitability, and 871.88 km^2^ as highly suitability. Under the SSP8.5 scenario, the total suitable area is 17,491.54 km^2^, with 8987.49 km^2^ of low suitability, 3823.79 km^2^ of moderately suitability, and 4680.27 km^2^ of highly suitability. For 2070, under the SSP4.5 scenario, the total suitable area for *E. pinifolius* is projected to be 19,054.77 km^2^. Of this, 10,128.89 km^2^ is classified as lowly suitable habitat, 3952.99 km^2^ as moderately suitable habitat, and 4972.89 km^2^ as highly suitable habitat. Under the SSP8.5 scenario, the total suitable area is expected to be 17,673.79 km^2^, with 12,143.89 km^2^ categorized as lowly suitability habitat, 4602.41 km^2^ as moderately suitable habitat, and 927.50 km^2^ as highly suitable habitat (Table 3, Figure 9).

Compared to the current habitat suitability, future scenarios under both SSPs show an overall increase in total suitable area, although the distribution of suitability levels varies across years and scenarios. In 2050, under SSP4.5, lowly suitable (+32.5%) and moderately suitable (+4.2%) areas increase, while highly suitable areas decline (−79.8%). Under SSP8.5, highly suitable areas slightly increase (+8.2%), whereas moderately (−7.0%) and lowly suitable (−3.5%) areas decline. By 2070, SSP4.5 predicts gains in highly suitable (+14.9%) and lowly suitable (+8.8%) areas, with a slight decrease in moderately suitable areas (−3.8%). In SSP8.5, highly suitable areas again decrease substantially (−78.6%), while moderately (+12.0%) and lowly suitable (+30.4%) areas expand. Unsuitable areas remain nearly unchanged across all scenarios (Table 4, Figure 9).

**TABLE 4:** Percentage changes in habitat suitability classes of *E. pinifolius* between current and future climate scenarios (SSP4.5 and SSP8.5) for 2050 and 2070.

Climatic period	Scenarios	Change (%) compared to the current suitability			
		Highly suitable	Moderately suitable	Lowly suitable	Unsuitable
Current	—	—	—	—	—
2050	SSP4.5	−79.8	+4.2	+32.5	+0.02
	SSP8.5	+8.2	−7.0	−3.5	+0.02
2070	SSP4.5	+14.9	−3.8	+8.8	−0.12
	SSP8.5	−78.6	+12.0	+30.4	+0.01

## 4 Discussion

*E. pinifolius* habitat suitability in Ethiopia was predicted with a high degree of accuracy using the MaxEnt model. The model exhibited excellent predictive performance, with an AUC of 0.985, indicating strong reliability in forecasting the species' potential habitats and distribution patterns across the region. This high performance is consistent with both regional and global applications of MaxEnt, further demonstrating its robustness for modeling habitat suitability under current and future climate scenarios. For instance, Mekasha et al. (2026) applied MaxEnt to *Commiphora africana* in Ethiopia and reported strong model predictions that inform conservation priorities under future climate change scenarios. Similarly, a MaxEnt study on *Oxytenanthera abyssinica* in northern Ethiopia yielded AUC values exceeding 0.90, placing the model's performance in the “excellent” category and corroborating its utility for predicting habitat shifts under climate change (Gebrewahid et al. 2020). By contrast, Gebrehiwot et al. (2022) modeled the distribution of *Podocarpus falcatus* with a moderate AUC of ~0.78, illustrating how species‐specific ecology and data quality can influence model accuracy while still providing useful insights for landscape‐scale restoration planning. At a global scale, Li, Geng, et al. (2025); Li, Zhang, et al. (2025) used MaxEnt to project macadamia habitat suitability under future climate scenarios and achieved an average AUC of 0.98, highlighting the model's capacity to reliably discriminate suitable versus unsuitable areas across diverse biogeographic contexts. Collectively, these studies support MaxEnt's effectiveness when calibrated with robust occurrence data and pertinent bioclimatic predictors, reinforcing its role in guiding conservation and management strategies.

Mean Temperature of the Driest Quarter (BIO9) is the most influential variable shaping the habitat suitability of *E. pinifolius*. In our study, *E. pinifolius* showed optimal suitability at moderate temperatures, around 10°C during the driest quarter. Suitability remained low at temperatures below ~5°C, while at temperatures above 30°C, suitability markedly declined (Figure 7, E), which may be due to moisture limitations affecting establishment and survival. Temperature during the driest period is likely a critical ecological constraint, as plants in montane ecosystems experience combined stress from limited moisture availability and temperature fluctuations (Brochmann et al. 2022; Li, Geng, et al. 2025; Li, Zhang, et al. 2025). These factors strongly influence plant physiology, phenology, and seedling establishment, thereby shaping species distribution patterns (Harrison and Prentice 2003; Körner 2007). Recent studies on alpine and montane plants similarly highlight temperature‐related variables as major drivers of species distributions because they regulate metabolic processes, growth, and regeneration (Zu et al. 2024).

Altitude also played a key role in determining suitable habitats. Elevation acts as an integrative environmental gradient influencing temperature, precipitation, solar radiation, soil development, and moisture availability (Hijmans et al. 2005; Graham and Hijmans 2006). For many alpine and montane species, elevational gradients strongly constrain species distributions because they define ecological niches characterized by specific climatic conditions. Elevation and temperature gradients are among the most important factors shaping alpine plant distribution and range limits (Felkel et al. 2023; Zu et al. 2024). The predicted spatial distribution of suitable habitats for *E. pinifolius* was mainly concentrated in the mountainous regions of the Ethiopian highlands, particularly in the northern, northwestern, and central parts of the country. This spatial pattern is consistent with previous botanical records, indicating that the species occurs predominantly in Afroalpine vegetation zones (Vivero et al. 2005). Afroalpine ecosystems are generally restricted to elevations between approximately 3200 and 4533 m above sea level and are characterized by unique climatic conditions, including low temperatures, high solar radiation, and strong diurnal temperature fluctuations (Hedberg 1995). These harsh environmental conditions restrict the distribution of many plant species but provide specialized habitats for cold‐adapted taxa. A study conducted by Gebrehiwot et al. (2020) reported that in the Afroalpine vegetation of the Abune Yosef massif, *Euryops pinifolius* forms a dominant shrub layer at 3834–4168 m a.s.l., co‐occurring with herbaceous species such as *Kniphofia foliosa*, *Cardamine hirsuta* , *Artemisia abyssinica*, *Festuca macrophylla*, and *Senecio farinaceus*. These communities persist under extremely high‐elevation conditions, highlighting the species' adaptation to stress‐prone environments and its narrow ecological niche.

Under future climate change scenarios (SSP4.5 and SSP8.5 for 2050 and 2070), the model predicts an increase in climatically suitable habitats for *E. pinifolius*. This projected expansion may be related to the upward shift of climatic niches as global temperatures increase. Similar trends have been reported in other montane ecosystems, where warming temperatures allow plant species to colonize higher elevations or newly suitable environments (Lenoir et al. 2008; Gao et al. 2022). Climate change may cause shifts in alpine vegetation distributions toward higher altitudes or latitudes as species track suitable climatic conditions (Bueno de Mesquita et al. 2024; Reichmuth et al. 2025). Although detailed physiological studies on *E. pinifolius* are lacking, its occurrence across a relatively broad elevational range suggests some level of ecological tolerance to environmental variability. This adaptability may be supported by physiological plasticity or interactions with associated soil microbial communities that help plants cope with environmental stress in alpine ecosystems (Hou et al. 2024). Similar adaptive strategies have been observed in other Afroalpine taxa, including species of Lobelia, which exhibit specialized morphological and physiological traits allowing them to survive in extreme highland environments (Knox and Palmer 1995).

Under the moderate‐emission SSP4.5 scenario, highly suitable habitats for *E. pinifolius* decline by 2050 but recover by 2070, reflecting initial habitat loss within its current range followed by new suitable areas at higher elevations (Thuiller et al. 2011). Under the high‐emission SSP8.5 scenario, suitable habitats increase slightly by 2050 but decline sharply by 2070, as extreme warming exceeds physiological limits (Chen et al. 2011; IPCC 2021). A study by Dagnachew et al. (2023) showed that *E. pinifolius* seeds maintain high viability under cold and temperature‐variable conditions, which may support persistence and colonization of newly suitable habitats under moderate warming, yet extreme temperatures projected under SSP8.5 could limit successful establishment despite this trait. These findings highlight that moderate warming may temporarily facilitate range shifts in montane plants, whereas extreme climate scenarios pose a greater risk of habitat loss.

## 5 Limitations and Future Research Directions

This study provides an important step toward understanding the potential future distribution of a threatened and culturally significant species under climate change. However, several limitations should be considered. Although the modeling framework focused on abiotic drivers, species distributions are also shaped by complex ecological and evolutionary processes. Interactions with pollinators (Inouye 2020; Richman et al. 2020), competition with other plant species (Anthelme and Dangles 2012), and eco‐evolutionary dynamics (Hamann et al. 2021) can significantly influence range shifts. In addition, integrating genotype, phenotype, and behavioral traits has been identified as critical for predicting species' responses to environmental extremes (Buckley et al. 2023), while neighboring species interactions may further affect survival and adaptation under changing climates (Damschen 2022).

This study's reliance on a single global circulation model (HadGEM2‐ES) may have limited the robustness of the forecasts, as different emission scenarios and climate models can yield different outcomes (Zhang et al. 2019). Future research should therefore incorporate multiple climate models and emission pathways to improve projection reliability. Similarly, the use of a single modeling algorithm (MaxEnt), despite the availability of alternative approaches within the Biomod2 framework, may introduce algorithm‐specific bias. Adopting ensemble modeling techniques that integrate multiple algorithms would enhance predictive performance and reduce uncertainty. The occurrence data, derived from GBIF databases, may also be subject to spatial sampling bias. Despite applying spatial filtering and bias correction, these limitations can influence predictions, particularly in under‐sampled regions. Field‐based validation through targeted surveys in newly identified suitable habitats would help improve model accuracy and reliability.

Finally, even where climatic suitability increases, anthropogenic pressures, including agricultural expansion, deforestation, land degradation, and human settlement may restrict habitat availability and connectivity, especially in the Ethiopian highlands (Getachew et al. 2020; Tefera et al. 2022). Addressing these challenges requires integrating socio‐ecological dimensions into species distribution models. Incorporating local and indigenous knowledge through co‐produced research approaches can provide valuable historical and ecological insights (Steger et al. 2020).

## 6 Implications for Conservation Plans

We propose the following conservation measures based on our findings. First, habitats that remain suitable in all future scenarios may be vital refuges for *E. pinifolius* conservation efforts. Adaptive management will be informed by regular monitoring of these refugia, enabling early detection of changes in species performance. Although future climate scenarios suggest expansion of suitable areas, the ability of *E. pinifolius* may be constrained by land‐use patterns, fragmentation, agriculture, and overgrazing (Friis et al. 2010; Gebrehiwot et al. 2020; Getachew et al. 2020). Nevertheless, these areas offer opportunities for targeted reforestation and habitat restoration, especially in high and moderate suitability zones. The need to incorporate biodiversity conservation into regional land management policy is emphasized by highlighting highland areas in northern, northwestern, and central Ethiopia as important refugia. Incorporating local communities and indigenous knowledge alongside scientific modeling can further enhance conservation effectiveness, providing insights into historical species distributions, ecosystem dynamics, and potential responses to environmental change (Steger et al. 2020). Future research should refine predictive models by including species interactions, land‐use dynamics, and local ecological knowledge to better guide conservation planning for *E. pinifolius* and other Afroalpine species.

## 7 Conclusions

Species distribution modeling (SDM) is a valuable tool for assessing the impact of climate change on species that require monitoring and management. Our model identified altitude and mean temperature of the driest quarter as the most influential environmental factors for predicting the distribution of *Euryops pinifolius*, reflecting the species' preference for high‐elevation, cooler, and seasonally dry microclimates. Vegetation cover and precipitation‐related variables also contributed to shaping habitat suitability, indicating that both climatic and local environmental factors limit the species' distribution. The results show that suitable habitat for *E. pinifolius* is largely concentrated in the Ethiopian highlands, particularly in the central, northern, and northwestern regions. These areas face multiple future threats, including climate change, habitat fragmentation, and land‐use change, which could reduce the extent and connectivity of suitable habitats. Understanding future predicted distribution can be used in conjunction with socio‐economic factors and indigenous knowledge to better inform conservation planning.

## Author Contributions

**Liyew Birhanu:** conceptualization (equal), data curation (equal), formal analysis (lead), investigation (equal), methodology (equal), resources (equal), software (equal), validation (equal), visualization (equal), writing – original draft (lead), writing – review and editing (equal). **Heiko Balzter:** conceptualization (equal), formal analysis (equal), funding acquisition (lead), investigation (equal), methodology (equal), project administration (lead), resources (equal), software (equal), supervision (lead), validation (equal), visualization (equal), writing – review and editing (equal). **Laura Basell:** conceptualization (equal), investigation (equal), methodology (equal), resources (equal), validation (equal), visualization (equal), writing – review and editing (equal). **Moya Burns:** conceptualization (equal), investigation (equal), methodology (equal), resources (equal), validation (equal), visualization (equal), writing – review and editing (equal). **Wale Arega:** conceptualization (equal), data curation (equal), resources (equal), visualization (equal), writing – review and editing (equal). **Yilkal Gebeyehu:** formal analysis (equal), resources (equal), writing – review and editing (equal).

## Funding

This research was supported by University of Leicester, through the visiting research fellowship. H.B. was supported by the Natural Environment Research Council of the UK through the National Centre for Earth Observation. L.B. was supported by a British Academy Mid‐Career Fellowship Reference (MFSS24\240089).

## Conflicts of Interest

The authors declare no conflicts of interest.

## Supporting information


**Data S1:** Supporting Information.

## Data Availability

The datasets used in this study are freely available from the following sources: Presence records of the 151 *Euryops pinifolius* are given in Table S1. Bioclimatic data: WorldClim (https://www.worldclim.org).
