# Supplementary material for: Climate‐Driven Habitat Suitability Modeling for the Vulnerable Species Euryops pinifolius A. Rich in Ethiopia: Implications for Conservation
Source: Ecol Evol. 2026 Apr 30;16(5):e73566. doi: 10.1002/ece3.73566 (PMC13130346; doi:10.1002/ece3.73566)
Supplement: Supplementary file 1 — Data S1: Supporting Information. [file ECE3-16-e73566-s001.pdf]

: Presence records of the 151 Euryops pinifolius are given in Table S1

Species	Longitude	Latitude
Euryops pinifolius	37.94135	10.67655
Euryops pinifolius	37.68333	10.77743
Euryops pinifolius	37.75021	10.77993
Euryops pinifolius	37.84277	10.71527
Euryops pinifolius	37.90305	10.75333
Euryops pinifolius	37.9761	10.73555
Euryops pinifolius	37.96222	10.76472
Euryops pinifolius	37.83333	10.76638
Euryops pinifolius	37.8961	10.70361
Euryops pinifolius	37.50388	10.98499
Euryops pinifolius	37.73054	10.08305
Euryops pinifolius	37.78944	10.58444
Euryops pinifolius	37.81333	10.61111
Euryops pinifolius	37.8411	10.63444
Euryops pinifolius	37.73333	10.7811
Euryops pinifolius	37.71611	10.74583
Euryops pinifolius	73.81138	10.76832
Euryops pinifolius	37.79833	10.73277
Euryops pinifolius	37.75666	10.72804
Euryops pinifolius	37.75666	10.72804
Euryops pinifolius	37.82193	10.69888
Euryops pinifolius	37.76832	10.68388
Euryops pinifolius	37.78471	10.68583
Euryops pinifolius	37.77971	10.65611
Euryops pinifolius	37.77027	10.63805
Euryops pinifolius	37.75944	10.61472
Euryops pinifolius	37.8361	10.62527
Euryops pinifolius	37.84416	10.68138
Euryops pinifolius	37.94138	10.67166
Euryops pinifolius	37.84333	10.75555
Euryops pinifolius	37.88444	10.64499
Euryops pinifolius	37.87277	10.61611
Euryops pinifolius	37.9608	10.76222
Euryops pinifolius	38.0186	13.23416
Euryops pinifolius	38.02554	13.22804
Euryops pinifolius	38.02888	13.22249
Euryops pinifolius	38.04471	13.23527
Euryops pinifolius	38.06527	13.23082
Euryops pinifolius	38.09499	13.255
Euryops pinifolius	38.21916	13.24721
Euryops pinifolius	38.155	13.24333
Euryops pinifolius	38.13777	13.20611
Euryops pinifolius	38.08721	13.22138
Euryops pinifolius	38.30555	13.34916
Euryops pinifolius	38.36444	13.2361
Euryops pinifolius	38.44138	13.2075

Euryops pinifolius	38.51305	13.23333
Euryops pinifolius	38.14221	13.20833
Euryops pinifolius	39.16277	12.10388
Euryops pinifolius	39.17693	12.14833
Euryops pinifolius	39.09055	12.04194
Euryops pinifolius	39.39194	12.03999
Euryops pinifolius	39.37888	11.74527
Euryops pinifolius	39.46361	11.5
Euryops pinifolius	39.50972	10.93333
Euryops pinifolius	39.72471	9.75694
Euryops pinifolius	39.73249	9.81916
Euryops pinifolius	39.73027	9.79721
Euryops pinifolius	39.72749	9.71361
Euryops pinifolius	39.8	10.3711
Euryops pinifolius	38.95972	10.87999
Euryops pinifolius	39.39027	9.30222
Euryops pinifolius	39.44221	11.03944
Euryops pinifolius	39.34694	10.91527
Euryops pinifolius	39.79555	10.305
Euryops pinifolius	38.41333	13.27243
Euryops pinifolius	39.76666	10.51666
Euryops pinifolius	37.85	10.53333
Euryops pinifolius	37.82566	10.656
Euryops pinifolius	37.83916	10.63816
Euryops pinifolius	37.822	10.6575
Euryops pinifolius	37.8356	10.642
Euryops pinifolius	37.805	10.595
Euryops pinifolius	39.33333	11.9
Euryops pinifolius	37.85	10.53333
Euryops pinifolius	39.68333	7.08333
Euryops pinifolius	39.65	7.05
Euryops pinifolius	39.65	7.06667
Euryops pinifolius	39.9	7.1
Euryops pinifolius	38.2	13.3
Euryops pinifolius	39.71388	6.8811
Euryops pinifolius	39.32777	6.79221
Euryops pinifolius	39.39805	7.94583
Euryops pinifolius	39.46333	7.84916
Euryops pinifolius	39.71804	7.11721
Euryops pinifolius	39.45277	6.72582
Euryops pinifolius	39.2361	6.86667
Euryops pinifolius	39.09444	6.84527
Euryops pinifolius	39.91333	6.80527
Euryops pinifolius	39.73777	7.10111
Euryops pinifolius	39.72221	7.25
Euryops pinifolius	39.64666	7.11971
Euryops pinifolius	39.24861	6.98944
Euryops pinifolius	39.75972	6.88389

Euryops pinifolius	39.41667	6.66667
Euryops pinifolius	39.33333	7.98333
Euryops pinifolius	38.10583	13.26977
Euryops pinifolius	38.11882	13.28798
Euryops pinifolius	38.19429	13.26287
Euryops pinifolius	39.73333	9.81667
Euryops pinifolius	39.66667	9.75
Euryops pinifolius	39.7	9.8
Euryops pinifolius	39.79277	9.82584
Euryops pinifolius	39.56167	9.28889
Euryops pinifolius	39.65083	10.38167
Euryops pinifolius	39.71056	10.57889
Euryops pinifolius	39.71278	10.63777
Euryops pinifolius	39.3925	10.96056
Euryops pinifolius	39.46945	10.94861
Euryops pinifolius	39.40278	10.03333
Euryops pinifolius	39.28361	10.98694
Euryops pinifolius	39.17667	11.04889
Euryops pinifolius	39.18223	12.14277
Euryops pinifolius	39.18444	12.15222
Euryops pinifolius	39.05083	11.83639
Euryops pinifolius	39.35667	11.945
Euryops pinifolius	39.39333	11.93805
Euryops pinifolius	38.72695	9.68611
Euryops pinifolius	39.72417	9.72639
Euryops pinifolius	39.53861	9.30361
Euryops pinifolius	39.1975	11.77334
Euryops pinifolius	38.08611	11.80306
Euryops pinifolius	38.20417	11.64972
Euryops pinifolius	38.32223	11.6825
Euryops pinifolius	38.22473	11.80444
Euryops pinifolius	38.29333	11.74416
Euryops pinifolius	39.8	10.66667
Euryops pinifolius	38.22778	11.836111
Euryops pinifolius	38.23889	11.71417
Euryops pinifolius	38.14722	11.72334
Euryops pinifolius	38.41528	11.71806
Euryops pinifolius	38.50833	11.67973
Euryops pinifolius	38.37639	11.71889
Euryops pinifolius	38.26583	11.77473
Euryops pinifolius	38.2125	11.74444
Euryops pinifolius	38.22584	11.61111
Euryops pinifolius	38.18666	11.66889
Euryops pinifolius	39.18333	12.13333
Euryops pinifolius	38.24861	13.14277
Euryops pinifolius	38.25611	13.29805
Euryops pinifolius	38.50694	13.23944
Euryops pinifolius	38.50083	13.3875

Euryops pinifolius	38.14777	13.14527
Euryops pinifolius	38.36917	13.08694
Euryops pinifolius	38.23361	13.26111
Euryops pinifolius	38.20306	13.34639
Euryops pinifolius	38.51	13.22445
Euryops pinifolius	37.97973	13.20722
Euryops pinifolius	38.36611	13.2475
Euryops pinifolius	37.99055	13.19972
Euryops pinifolius	38.41333	13.26723
